# First experimental proof of Proton Boron Capture Therapy (PBCT) to enhance protontherapy effectiveness

**DOI:** 10.1038/s41598-018-19258-5

**Published:** 2018-01-18

**Authors:** G. A. P. Cirrone, L. Manti, D. Margarone, G. Petringa, L. Giuffrida, A. Minopoli, A. Picciotto, G. Russo, F. Cammarata, P. Pisciotta, F. M. Perozziello, F. Romano, V. Marchese, G. Milluzzo, V. Scuderi, G. Cuttone, G. Korn

**Affiliations:** 1 Istituto Nazionale di Fisica Nucleare- Laboratori Nazionali dei Sud, via S. Sofia, 62 Catania, Italy; 2 Physics Department, University of Naples Federico II, Naples, Italy; 3 INFN Naples Section, Complesso Universitario di Monte S. Angelo, Via Cintia, Naples, Italy; 4 Institute of Physics ASCR, v.v.i. (FZU), ELI-Beamlines Project, Na Slovance 2, Prague, 18221 Czech Republic; 5 Physics Department, University of Catania, via S. Sofia, 64 Catania, Italy; 6 Fondazione Bruno Kessler, Micro-Nano Facility, Via Sommarive 18, 38123 Povo-Trento, Italy; 7 Institute of Molecular Bioimaging and Physiology - National Research Council - (IBFM-CNR), Cefalù, (PA) Italy; 8 National Physical Laboratory, Acoustic and Ionizing Radiation Division, Teddington, TW11 0LW Middlesex United Kingdom

## Abstract

Protontherapy is hadrontherapy’s fastest-growing modality and a pillar in the battle against cancer. Hadrontherapy’s superiority lies in its inverted depth-dose profile, hence tumour-confined irradiation. Protons, however, lack distinct radiobiological advantages over photons or electrons. Higher LET (Linear Energy Transfer) ^12^C-ions can overcome cancer radioresistance: DNA lesion complexity increases with LET, resulting in efficient cell killing, i.e. higher Relative Biological Effectiveness (RBE). However, economic and radiobiological issues hamper ^12^C-ion clinical amenability. Thus, enhancing proton RBE is desirable. To this end, we exploited the p + ^11^B → 3α reaction to generate high-LET alpha particles with a clinical proton beam. To maximize the reaction rate, we used sodium borocaptate (BSH) with natural boron content. Boron-Neutron Capture Therapy (BNCT) uses ^10^B-enriched BSH for neutron irradiation-triggered alpha particles. We recorded significantly increased cellular lethality and chromosome aberration complexity. A strategy combining protontherapy’s ballistic precision with the higher RBE promised by BNCT and ^12^C-ion therapy is thus demonstrated.

## Introduction

The urgent need for radical radiotherapy research to achieve improved tumour control in the context of reducing the risk of normal tissue toxicity and late-occurring sequelae, has driven the fast-growing development of cancer treatment by accelerated beams of charged particles (hadrontherapy) in recent decades^1^. This appears to be particularly true for protontherapy, which has emerged as the most-rapidly expanding hadrontherapy approach, totalling over 100,000 patients treated thus far worldwide^2^. Wilson first proposed the use of energetic protons for cancer radiotherapy in 1946^3^. The primary motivation for investigation into this area was based on the physical properties of charged particles, which can deposit energy far more selectively than photons: through the inverted depth-dose profile described by the Bragg curve^4^, healthy tissues within the entry channel of the beam are spared of dose, while most of the dose is steeply confined at the end of the particle range (the so-called “Bragg peak”). This in principle enables the delivery of very high-dose gradients close to organs at risk, confining the high-dose area to the tumour volume. Despite the dearth of randomized trials showing an effective advantage of protons over photon-based radiotherapy^5,6^ and the ongoing debate over its cost-effectiveness^7^, the current phase I/II clinical results support the rationale of the approach, especially for deep-seated tumours localized in proximity of critical organs, and unresectable or recurrent tumours^8,9^. Cancer treatment by protons also remains the most attractive solution in the case of paediatric patients due to the significant reduction in the integral dose delivered to the patient^8^, even compared to newer photon techniques such as intensity modulated radiation therapy^10^. However, protons have been traditionally regarded as only slightly more biologically effective than photons^11^. In fact, the standard practice in protontherapy is to adopt an RBE (Relative Biological Effectiveness) value of 1.1 compared to photons in any clinical condition^12^, although such an assumption overlooks the increased RBE of low-energy protons^13–15^ disregarding recently unveiled peculiarities of proton radiobiology^8,16,17^.

The combination of ballistic precision with an increased ability to kill cells is the radiobiological rationale currently supporting the clinical exploitation of heavier particles such as fully stripped ^12^C-ions^18^, which present some advantages over protons^6,18^. Not only do they ensure a better physical dose distribution, due to less lateral scattering^19^, but they also result more biologically effective both *in vitro* and *in vivo* as a result of their higher Linear Energy Transfer (LET)^11,20,21^. In fact, densely ionizing radiation tracks cause more spatio-temporally contiguous and complex lesions at the DNA level, comprising DNA double-strand breaks and damaged bases, which are highly clustered in nature^22–24^. This impairs cellular ability for correct repair^25^ and decreases the dependence of radiosensitization upon the presence of oxygen, desirable features for eradication of resilient, hypoxic tumors^5,26^. Further potential radiobiological advantages include greater RBE for killing putatively radioresistant cancer stem cells^27^ and counteracting cancer invasiveness^28,29^, albeit the latter remains controversial^30^. Finally, low doses of high-LET radiation appear to elicit stronger immunological responses compared to low-LET radiation^16^.

On the other hand, complications related to nuclear fragmentation from the primary beam, along with a partial understanding of the consequences of the exposure of normal cells to high-LET radiation, and also considering the complexity and high costs associated with a ^12^C treatment facility, fueled research into exploring novel strategies with the aim to achieve alternative solutions for a localized increase of proton RBE.

One of such recently proposed approaches foresees the use of gold nanoparticles as protontherapy radiosensitizers^31^. The ability of particle radiation to stimulate favourable immunological responses represents another attractive solution as it has become increasingly evident that proton and photon irradiation differentially modulate systemic biological responses^8,17^.

In this work, we experimentally test for the first time the idea theoretically proposed by Do-Kun Y *et al*. in^32^, based on the use of the p + ^11^B → 3α nuclear fusion reaction^33,34^ to enhance proton biological effectiveness exclusively in the tumour region through the generation of short-range high-LET alpha particles, thus being of potential clinical worth. Cells were irradiated with a clinical proton beam in the presence of sodium borocaptate (NA_2_B_12_H_11_SH or “BSH”), which is a common agent clinically used in BNCT in its ^10^B-enriched form to selectively deliver given boron concentrations in cancer cells^35^. BNCT requires thermal neutrons to trigger the reaction where two charged particles (one alpha of 1.77 MeV and one ^7^Li ion of 1.015 MeV) with a positive Q value of 2.792 MeV are produced^36^. In order to maximize the p + ^11^B → 3α fusion rate, we used BSH with natural occurring boron isotopic abundance (i.e. about 80% ^11^B and 20% ^10^B). We observed a significant increase in proton-induced cytogenetic effects, both in terms of cell death, assessed as loss of proliferative potential by the clonogenic assay, and of induction of DNA damage. The latter was studied by chromosome aberrations revealed by Fluorescence *In Situ* Hybridization (FISH) painting techniques. Specifically, the markedly higher frequency of complex-type chromosome exchanges (a typical cytogenetic signature of high-LET ionizing radiation, see ref.^37^, which was found among boron-treated cells compared to proton-irradiated cells in the absence of BSH, points to alpha particles generated in the nuclear fusion reaction as being responsible for the measured enhancement of proton biological effectiveness. These findings, therefore, yield important implications for an enhanced cancer protontherapy.

## Results

### Experimental approach

The proton-boron nuclear reaction considered in this work is usually formalized as p + ^11^B —> 3α. It has a positive Q-value (8.7 MeV) and is often referred to as “proton-boron fusion reaction” since the incident proton is completely absorbed by the ^11^B nucleus. This reaction has garnered interest since the 1930s^33,34^ because of the process ability to produce copious numbers of alpha particles in an exothermic reaction.

According to the most recent studies and interpretations, p + ^11^B —> 3α can be described as a two-step reaction (involving ^12^C and ^8^Be nuclei excitation) with a main resonance occurring at a 675 keV (center of mass energy) and where the maximum cross section of 1.2 barn is measured^38,39^. A more detailed description of the reaction is reported in *Methods*. The emitted alpha particles exhibit a wide energy spectrum with a predominant energy around 4 MeV^40^. Such a reaction has been considered very attractive for the generation of fusion energy without producing neutron-induced radioactivity^41,42^.

Such a nuclear reaction may be even more useful as it could play a strategic role in medical applications improving the effectiveness of protontherapy. The potential clinical use of the p-^11^B reaction has been thus far only investigated and validated by means of Monte Carlo simulations^32,43^, with preliminary experimental work on its imaging potentialities^44,45^ being also performed. In this paper, we experimentally implement for the first time this innovative approach in a clinical scenario by measuring the biological effects as a direct consequence of the p-^11^B reaction.

**Figure 1:**
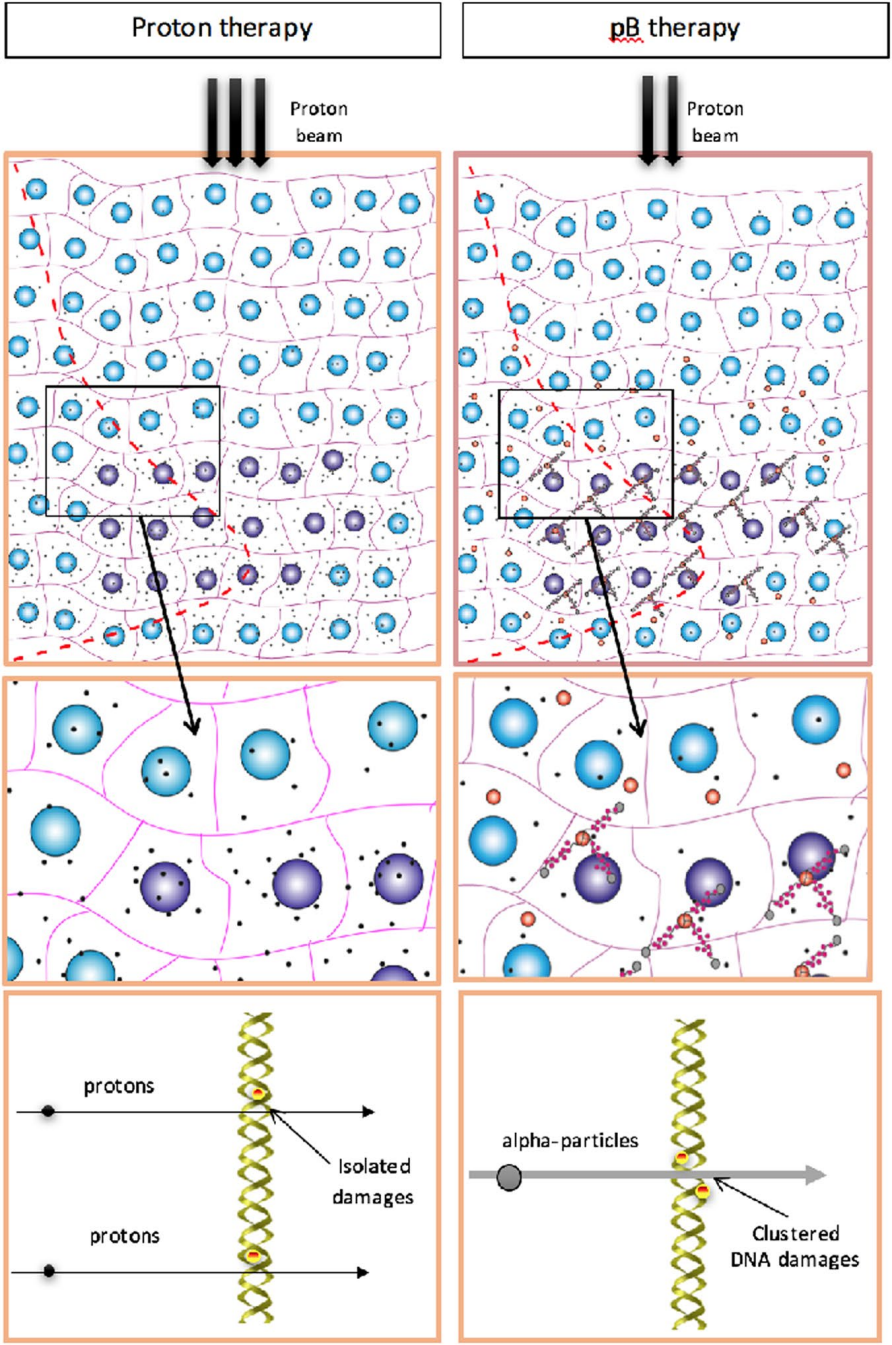
Schematic representation of “conventional” radiotherapy by low-LET proton beams (**A**) and the rationale of boron-enhanced protontherapy (**B**). Whereas in **A**) the incident proton beam mainly results in isolated, mostly repairable DNA breaks, the extremely localized emission of high-LET radiation produced by the proton-boron fusion in the Bragg peak region causes irreparable clustered DNA damage, similar in nature to that induced by ^12^C ions, hence the expected increase in effectiveness at tumor cell killing.

Besides the advantage of using a neutron-free nuclear fusion reaction, the relevance of this method stems from the fact that the p + ^11^B → 3α cross section becomes significantly high at relatively low incident proton energy, i.e. around the Bragg peak region. As schematically depicted in Fig. 1, a proton beam as conventionally used in protontherapy is drastically slowed down across the tumour (the Bragg peak region). Thus, most of its energy (dose) is delivered to the tumour cells. Under the assumption that a given concentration of ^11^B nuclei is present preferentially, but not exclusively, in the tumour, the arrival of slow protons could trigger a series of fusion events generating several alpha particles that are localized in the tumour region. In fact, most of the alpha particles generated in the proton-boron reaction have an average range in water of less than 30 μm, thus comparable with the typical cell size. Hence, even if such particles are mainly produced outside the cell cytoplasm due to sub-optimal boron uptake, the probability that they would reach the nucleus and damage the DNA remains very high. Moreover, even if a non-negligible concentration of ^11^B nuclei is present in the healthy tissues surrounding the tumour, the number of fusion events (i.e. generated alpha particles), would be relatively low, or completely absent, due the non-favourable incident proton energy spectrum away from the tumour region. This would lead to a more biologically effective particle dose localization, higher than the one currently achievable with conventional protontherapy, thus to a more efficient treatment in terms of an enhancement in cancer cell lethality, especially because of the clustered nature of the DNA damage, which is caused by the high-LET alpha particles emitted in the tumour region. Hence, protontherapy could acquire the benefits of an enhanced efficiency in cancer cell killing, moving close to ^12^C ion hadrontherapy but without the above-mentioned complications of the latter.

The ballistic advantage granted by the inverted dose-depth profile of charged particles is such that in protontherapy most of the dose is released mainly in the tumor region (upper panel), cancer cells being represented here by purple circles and damaging events by black dots (A): proton-induced damage is similar to that imparted to DNA by photons, consisting mainly of isolated lesions (middle and lower panels). If cancer cells are loaded with ^11^B-delivering agents (middle panel, B), as is the case with ^10^B-enriched compounds in BNCT, unrepairable DNA clustered lesions will be also produced by the high-LET alpha particles generated by the p-^11^B nuclear fusion reaction (lower panel). This in turn leads to a Dose Modifying Factor (DMF) for cancer cell killing while maintaining beneficial sparing of surrounding healthy tissues (middle panel). Furthermore, for a given clinical case, such higher DMF can potentially allow to reduce the overall dose delivered to the patient (lower total number of protons used in the number of fractions) compared to a standard treatment without the presence of ^11^B-delivering agents in the tumor.

### BSH enhances cancer cell death following proton-irradiation

**Figure 2:**
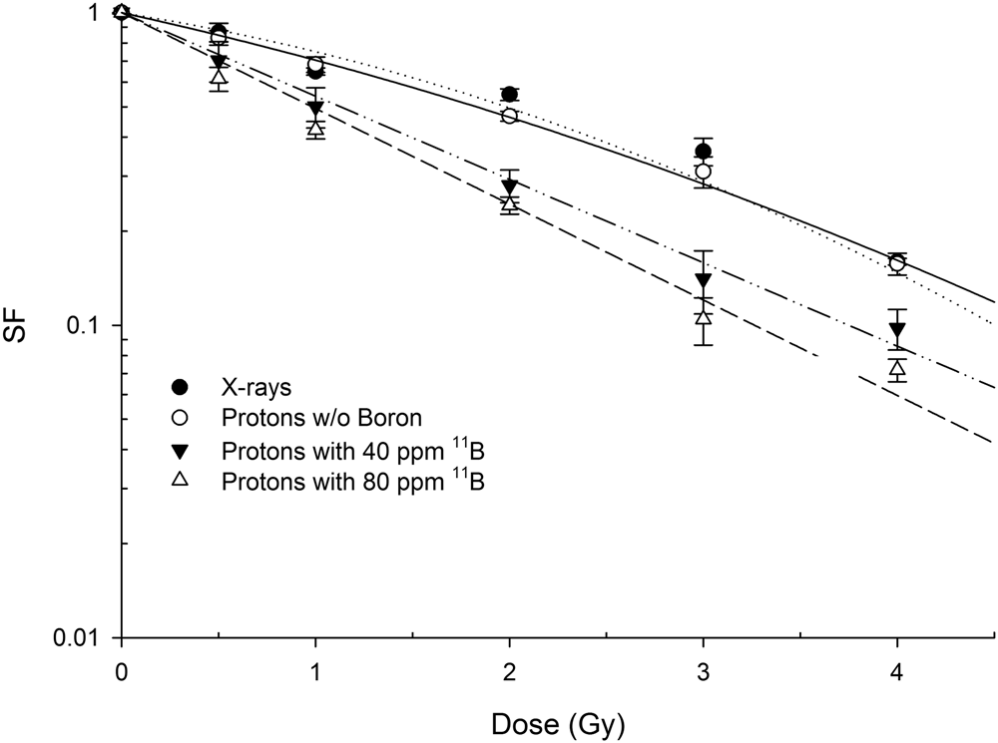
Boron-mediated increase in proton irradiation-induced cell death. Clonogenic dose response curves of prostate cancer cells DU145 irradiated with therapeutic protons in the presence or absence of BSH at mid-SOBP. Data are weighted mean values plus standard error from four independent experiments in the case of proton irradiation in the absence of BSH (open circles) and in the presence of the compound at the highest concentration used (80 ppm, open triangles). Two experiments were performed with cells irradiated in the presence of 40 ppm of ^11^B. X ray-irradiation survival data are also shown for comparison.

To test whether the p + ^11^B → 3α reaction results in an enhancement of cell killing by therapeutic proton beam irradiation, cells from the human prostate cancer line DU145 were irradiated with graded doses at the middle position of the 62-MeV clinical Spread-Out Bragg Peak (SOBP) using the proton beam at the superconducting cyclotron of the INFN-LNS facility (Catania, Italy). Irradiations were performed in the presence of two concentrations of BSH (see *Methods* for details on the irradiation set-up and BSH pre-treatment). As a control, prostate cancer DU145 cells grown and irradiated without BSH were used. The considered BSH concentrations were equivalent to 40 ppm (parts per million) and 80 ppm of ^11^B. These were chosen based on values from the literature on the ^10^B-enriched BSH analogue used in BNCT in order to achieve the optimal intracellular ^10^B concentration^35,46,47^. In particular, similar boron-equivalent concentration ranges of another BNCT compound, BPA, had been previously used with the same cell line *in vitro*^48^. Boron treatment enhanced proton biological effectiveness resulting in a significant increase in the induction of cell death in DU145 cells as measured by loss of colony-forming ability. Cells that were irradiated after pre-treatment with, and in the presence of, boron-containing BSH exhibited a greater radiosensitivity in comparison with cells exposed to radiation alone: BSH-treated cells yielded a much steeper clonogenic dose-response curve than that obtained for cells grown and irradiated in BSH-free medium (Fig. 2). The clonogenic survival fraction SF following irradiation with protons alone was best fitted to a linear-quadratic function of dose, i.e. SF = exp (-α*D-β*D^2^), with data from proton irradiation in the presence of BSH exhibiting a purely exponential behaviour as a function of dose. Least-square fitting parameters are reported in Table 1.

**Table 1:** Cell killing dose-response fitting parameters.

	α (Gy^−1^)	β (Gy^−2^)
X ray irradiation	0.222 ± 0.062	0.064 ± 0.014
Proton irradiation in the absence of BSH	0.314 ± 0.022	0.035 ± 0.007
Proton irradiation with 40 ppm ^11^B	0.614 ± 0.069	—
Proton irradiation with 80 ppm ^11^B	0.705 ± 0.033	—

Calculated values for the α and β parameters as obtained from the fitting of experimental data by the linear-quadratic model for radiation-induced cell killing are reported. Statistically equivalent to zero β values were found for proton irradiation in the presence of BSH.

A slight yet not statistically significant effect due to boron concentration was observed. Based upon the measured survival dose-responses, a calculated DMF of 1.46 ± 0.12 was determined at the 10% survival level (DMF_10_). This indicated that the presence of the boron compound conferred a radiobiological advantage at reducing cell survival compared to proton irradiation alone. At the concentrations used, BSH was not cytotoxic since the proliferative potential of unirradiated cells as given by cellular plating efficiency was not affected by the presence of BSH (Table 2). This means that the measured enhancement of proton effectiveness at cell killing was not contributed to by cytotoxicity caused by the boron-containing compound *per se*.

**Table 2:** Cytogenotoxicity of BSH alone.

	Plating efficiency	Baseline CA frequency
No BSH	0.58 ± 0.02	0.027 ± 0.003
40 ppm ^11^B	0.61 ± 0.04	0.027 ± 0.003
80 ppm ^11^B	0.60 ± 0.04	0.023 ± 0.003

Plating efficiency (PE) values and total chromosome aberration (CA) yields in unirradiated DU145 prostate cancer cells (second column) and normal epithelial MCF-10A cells (third column), respectively, as a function of the amount of BSH. By definition, PE measures the survival of cells in the absence of radiation. Similarly, the recorded frequency of CAs in cells not exposed to radiation is referred to as baseline CA frequency. Data show lack of BSH-induced cyto- and genotoxicity at the used concentration.

### Dependence of BSH-mediated cell killing enhancement upon proton energy

**Figure 3:**
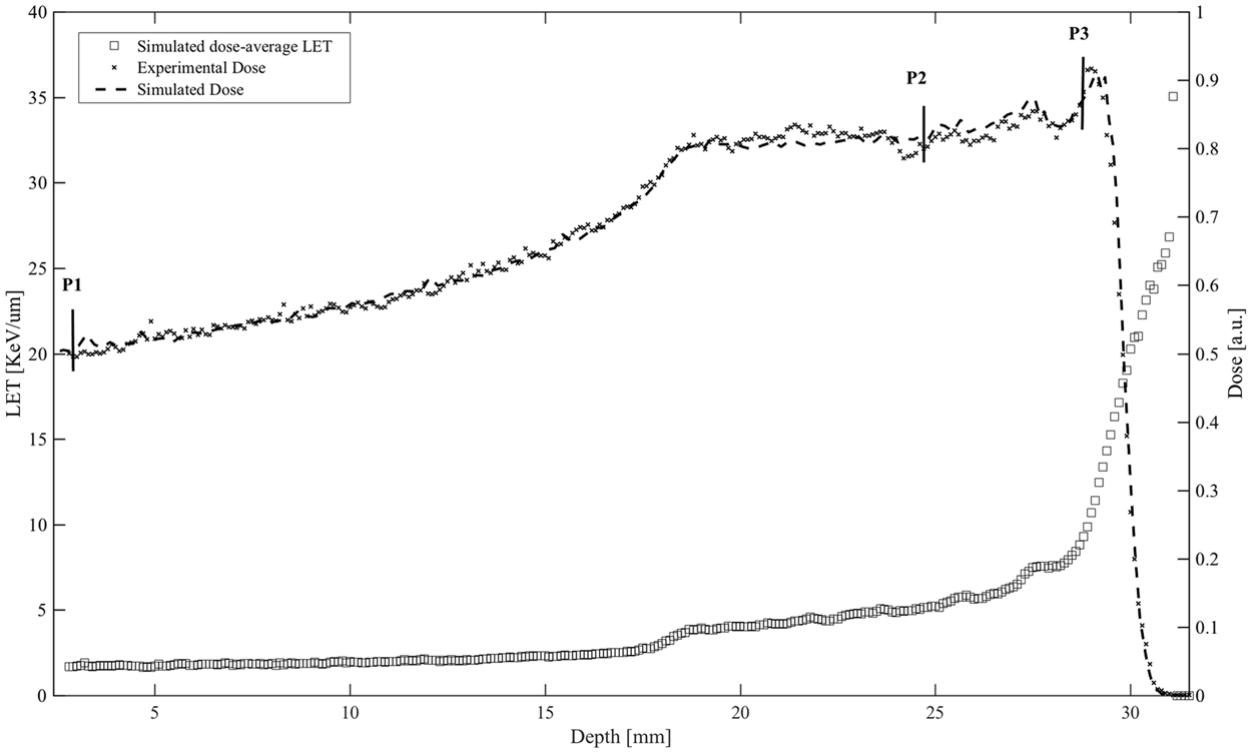
Cell irradiation along the proton SOBP. Measured dose and calculated LET profile for cellular irradiation at different positions along the clinical proton SOBP at INFN-LNS, Catania, Italy. Shown are the three depths along the SOBP at which cells were irradiated and the corresponding calculated LET values (open squares). Dose profiles as obtained by direct measurement by Markus chamber and by Monte-Carlo simulation.

Our working hypothesis is that the nuclear fusion p + ^11^B → 3α (p-B) reaction results in a significant increase in proton biological effectiveness due to the short-range high-LET alpha particles it produces. Such a reaction critically depends on the incident proton energy; hence, its radiobiological effectiveness can be expected to vary along the clinical proton SOBP. To verify this hypothesis, the induction of cell killing in the presence of the boron compound at the concentration of 80 ppm ^11^B (Fig. 2), was investigated irradiating the cancer DU145 cell line at the beam entrance (P1 position), at the SOBP distal end (P3 position), and at the middle of the SOBP (P2) as above reported (Fig. 3).

**Figure 4:**
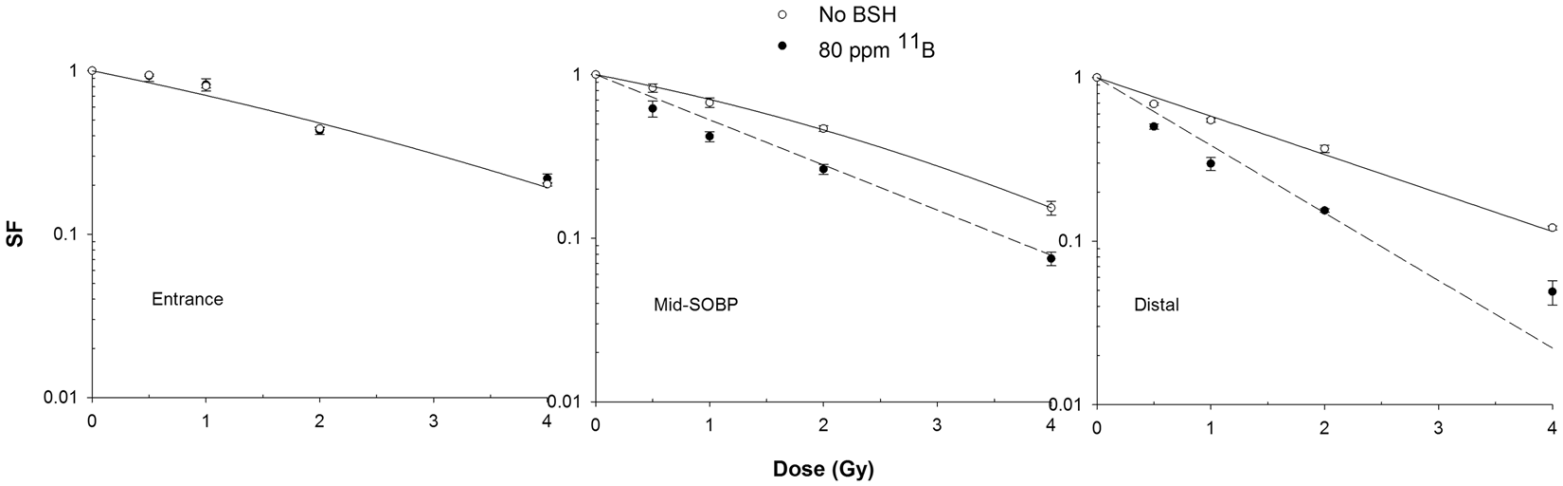
Clonogenic survival along the proton SOBP. Data shown here refer to dose-response curves obtained at positions P1, P2 and P3 as indicated in Fig. 3 along the clinical proton SOBP. Enhancement of cell killing due to the presence of the boron compound (black circles) is null at beam entrance (highest proton mean energy) while reaching its maximum at the distal end of the SOBP (lowest mean proton energies).

The panel in Fig. 4 shows the clonogenic survival dose-response curves derived from the three positions along the SOBP, in the absence or presence of BSH. In line with the expected variation in cell radiosensitivity along a clinical SOBP^14^, proton irradiation alone resulted in a progressive increase in cell killing from P1 to P3. Interestingly, data clearly show no effect of BSH at the beam entrance. A DMF of about 1.4 was confirmed at 10% cell survival at mid-SOBP: here, fitting parameters were α = (0.309 ± 0.022) Gy^−1^ and β = (0.040 ± 0.006) Gy^−2^ for proton irradiation without BSH and α = (0.653 ± 0.018) Gy^−1^ in the presence of 80 ppm ^11^B. At the distal end of the SOBP, however, BSH appeared to be even more effective with a recorded DMF of 1.75 ± 0.13: at this position, cell killing was best described by a pure exponential for both protons alone and protons in the presence of BSH, with values for the α parameter of (0.541 ± 0.027) Gy^−1^ and (0.952 ± 0.053) Gy^−1^. These experimental results, particularly the lack of a measurable effect due to the presence of ^11^B at beam entrance where the incident proton energy is the highest, confirm that the enhancement of biological effectiveness is caused by the occurrence of p-B nuclear fusion events, which have a higher probability (i.e. higher cross section) at relatively low energy (MeV level) of the incoming protons, i.e. towards the end of their range.

### BSH exacerbates proton irradiation-induced chromosome aberrations and enhances complex-type exchanges

**Figure 5:**
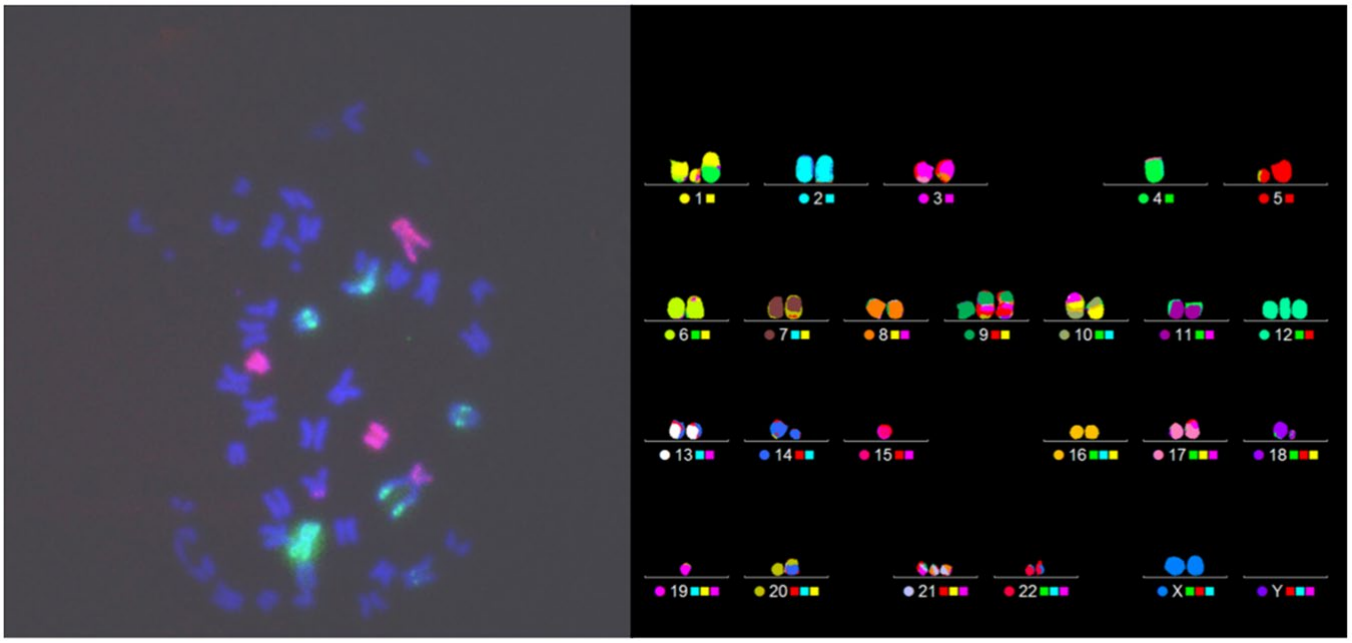
Analysis of proton irradiation-induced structural chromosome damage. Representative pictures of chromosome spreads from 4 Gy-proton irradiated cells treated with 80 ppm of ^11^B scored by conventional (left) and mFISH analysis (right), respectively. Both exhibit complex-type CAs. However, conventional FISH would, have detected neither the complex exchanges shown on the right that involves chromosomes 1, 10 e 19, nor the two translocations between chromosome 1 and 4 and between 14 e 20, as it paints just chromosomes 1 and 2. Using mFISH karyotyping analysis allows therefore more accurate measurement of DNA damage, especially of complex type.

Ionizing radiation can give rise to a wide spectrum of structural chromosome aberrations or CAs^49^. It is long-known that CAs are closely linked to clonogenic cell death^50^, hence an increase in cell killing due to the alpha particles produced by the p-B reaction ought to reflect in an increase in the yield of CAs. Furthermore, complex-type exchanges, defined as those rearrangements involving at least two chromosomes and generated by at least three breaks, are an archetypical feature of high-LET exposure^37,51^. Therefore, quantification of the proportion of complex-type chromosome exchanges was instrumental to unveil the action of high-LET alpha particles generated by the BSH-assisted p + ^11^B → 3α nuclear reaction. To this end, in addition to conventional FISH labelling (which is limited to painting two pairs of homolog chromosomes) a more comprehensive investigation was also carried out employing the multicolour(m)-FISH technique, which represents the method of choice when an accurate scoring of CAs, particularly of those of complex type, is required since it allows analysis of the whole karyotype. This is exemplified by Fig. 5 showing two cells from a sample irradiated with 4Gy of protons in the presence of 80 ppm ^11^B. One cell is conventionally FISH-painted, presenting with chromosome rearrangements of complex nature as several portions of the painted chromosomes are aberrantly joined with aspecifically stained chromosomes (appearing blue) and with themselves. The other cell has been subjected to mFISH analysis revealing a number of aberrations that would have gone undetected confining analyses to conventional FISH scoring.

Cancer cells are known to be genetically unstable; hence, they do not lend themselves to reliable assessment of radiation-induced DNA damage. Radiation-induced chromosome rearrangements would superimpose onto an elevated confounding frequency of baseline damage. Therefore, the non-tumorigenic breast epithelial MCF-10A cell line was chosen for scoring of radiation-induced CAs.

**Figure 6:**
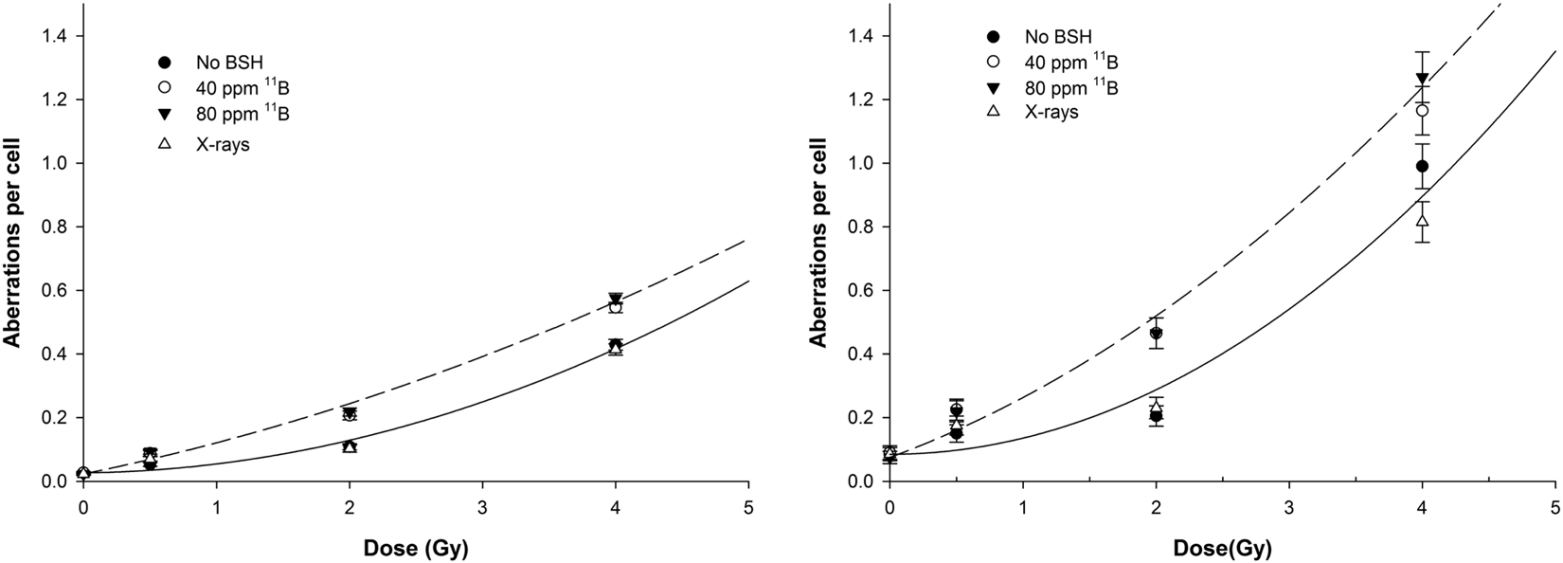
BSH-induced increased induction of chromosome aberrations following proton irradiation. The dose-dependent frequency of all chromosome exchanges scored by either conventional FISH painting (left) or m-FISH karyotyping (right) is shown for proton irradiation alone (black circles) and for proton irradiation in the presence of ^11^B at concentrations of 40 ppm (open circles) and 80 ppm (down triangles). X-ray data are also shown for comparison. In the interest of clarity, fitted curves are shown only for the highest boron concentration used (80 ppm, dashed line) and for irradiation with no boron compound (solid line). Data points correspond to the mean value measured in at least two independent experiments with standard errors of counts.

In cells not exposed to radiation, BSH *per se* did not cause significantly higher genotoxic damage compared to BSH-untreated cells, in agreement with the lack of cytotoxicity as measured by clonogenic assay (Table 2). Conversely, proton irradiation resulted in a higher yield of all CA types in cells treated with BSH compared to cells irradiated with protons in the absence of BSH (Fig. 6). As expected, the overall measured frequency of CAs raised for all irradiation conditions when using mFISH because of its greater sensitivity. Moreover, the boron-mediated enhancement of chromosomal damage is slightly amplified passing from conventional FISH scoring to mFISH analysis: the yield of CAs following 2Gy of protons in combination with BSH increased from about 0.22 to 0.47 aberrations per cell compared to about 0.11 and 0.20. Furthermore, when mFISH analysis was performed, measured CA frequency values “fan out” as dose increases showing a small effect due to ^11^B concentration at the highest proton dose, i.e. 4Gy, with 80 ppm of ^11^B appearing slightly more effective than 40 ppm. The yield of CAs following x-rays was identical to that measured after exposure to protons in the absence of BSH, in line with the observed lack of a significant difference in cell killing between x-rays and protons alone also seen in DU145 cancer cells (Fig. 2).

Aberration data were fitted to a linear –quadratic function of the type Y = Y_0_+α*D+β*D^2^ where the coefficient Y_0_ corresponded to the baseline CA frequency as reported in Table 2. As from mFISH analysis, following irradiation in the absence of BSH, no statistically significant value for the parameter α was found, while a value of β = (0.051 ± 0.026) Gy^−2^ could be derived; whereas α and β were (0.154 ± 0.066) Gy^−1^ and (0.034 ± 0.020) Gy^−2^ for proton irradiation in the presence of 80 ppm of ^11^B. Because of the purely quadratic nature of the dose-response curve for proton irradiation in the absence of BSH, no estimate for DMF_max_ could be derived as this is defined as the ratio of the linear components of the linear-quadratic dose-response curve by analogy with the concept of RBE^52^. DMF values were calculated for two levels of damage instead, that is 20 and 40 aberrations per 100 cells. In the case of 20 aberrations per 100 cells, the calculated DMF was about 2.1, whereas a DMF value of 1.6 was obtained for the level of 40 aberrations per 100 cells.

**Figure 7:**
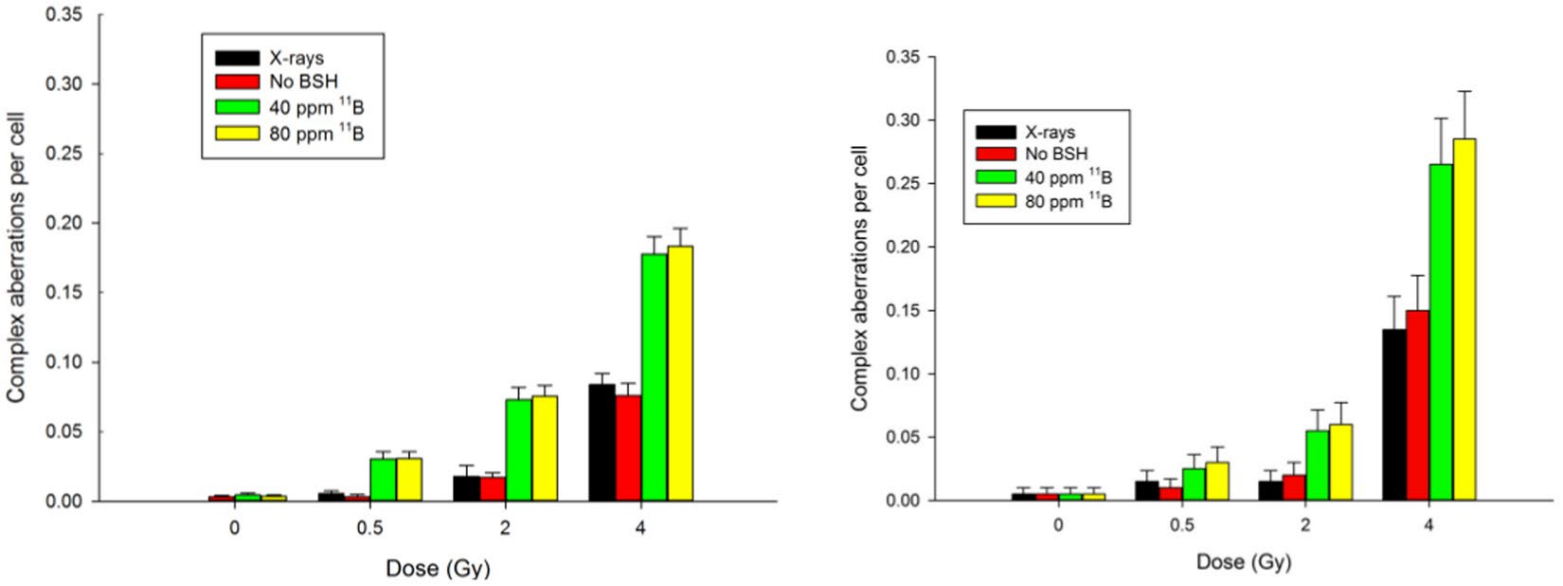
Induction of complex-type CAs. A greater proportion of complex chromosome rearrangements was found in cells irradiated with protons and treated with BSH than in cells subject to proton irradiation alone. Results of scoring with both conventional FISH techniques (left) are shown to highlight how mFISH (right) is much more efficient at detecting such type of CAs, which are the most significant biomarker of high-LET radiation exposure.

The most interesting result, however, came from the analysis of complex-type aberration exchanges. A markedly pronounced occurrence of complex-type exchanges was found in samples treated with BSH compared to cells that had been irradiated with protons in the absence of BSH (Fig. 7). After 0.5 Gy of protons, the frequency of complex CAs ranged between 0.025 and 0.028 for BSH-treated cells as opposed to less than 0.004 in the case of cells irradiated in the absence of BSH. These values dramatically increased with dose and remained consistently higher in the case of BSH-treated cells, reaching about 0.18 and 0.08 after 4 Gy of protons in the presence or the absence of BSH, respectively. Occurrence of complex-type exchanges following x-rays is also shown for comparison (Fig. 4) and does not differ from that measured for proton irradiation alone. Analysis by m-FISH confirmed such findings revealing how at high dose (4 Gy) conventional FISH scoring tends to underestimate the occurrence of complex exchanges, at which dose, as was the case for the total yield of aberrations, m-FISH unveils a slight BSH-concentration effect masked by conventional FISH painting.

Taken together, these results strongly support the notion that the presence of BSH results in a significant dose-modifying effect on proton irradiation, increasing cell lethality and DNA damage. In particular, the profoundly enhanced yield of complex-type CAs found following proton irradiation of BSH-treated cells points to damage brought about by high-LET radiation, thus consistent with the action of the alpha particles produced by the p-B fusion reaction.

## Discussion

The first experimental evidence of a significant enhancement of proton effectiveness at causing cytogenetic damage cells, through irradiation with a clinical proton beam in the presence of a compound with natural boron isotopic content (80% of ^11^B + 20% of ^10^B), is reported. The advantage of such a new methodology consists in the use of the p-B nuclear fusion reaction, which presents a high cross section for protons with energies in the range 0.1–10 MeV, i.e. around the Bragg peak. Such a reaction produces three alpha particles with a range comparable to the typical cells’ dimensions. BSH, one of the most commonly used boron-delivering agents in BNCT, served as natural boron carrier in our study. The rationale underlying BNCT consists in cancer irradiation by thermal neutrons, which results in a highly localized and mostly lethal targeting of cancer cells because of the very short range and, hence, of the high LET of the low-energy alpha particles generated in the reaction with the ^10^B atoms. However, despite numerous, carefully designed and generally promising clinical trials^53,54^, BNCT struggles to establish itself in clinical routine, both because of the intrinsically challenging quest for ideal carriers to deliver radiobiologically effective concentrations of boron to cancer cells, and because of the limited availability of thermal neutron sources^35,47^. On the other hand, albeit already being a clinical reality, external beam ^12^C-ion hadrontherapy, which is capable of delivering very biologically effective radiation doses with extremely high precision to the tumor target, is hampered by economic and radiobiological issues as discussed in the *Introduction* section.

Currently, the most widespread form of cancer treatment through accelerated charged particles is represented by protontherapy, which guarantees the same ballistic precision as ^12^C ion beams without the added complications. The disadvantage of protontherapy is, however, a low biological effectiveness at killing cells since protons are only about as effective as photons and electrons. The approach we have successfully tested in several experimental runs could represent an enormous step towards the ability to increase the biological efficacy of proton radiotherapy and therefore expand its use towards the treatment of radioresistant tumours by coupling the already favorable spatial characteristics of protons with the capacity to trigger the proposed reaction exclusively inside the tumor. In principle, this would enable the avoidance of the intrinsic uncertainties and enormous handling complications associated with the use of neutron beams in BNCT and could lead to an increase of proton biological effectiveness towards values closer to those exhibited by ^12^C-ions.

The biological effects of the p-B fusion reaction were investigated by measuring clonogenic cell death and chromosome aberrations (CAs) in a prostate cancer cell line (DU145) and in a non-tumorigenic epithelial breast cell line (MCF-10A), respectively. The latter was best suited to investigate chromosomal damage as opposed to genomically unstable cancer cells.

We found that proton irradiation-induced cellular lethality was greatly enhanced by irradiating cells that had been pre-treated with BSH. In particular, our results for survival of DU145 cells following low-LET proton irradiation alone are in line with those obtained at another protontherapy facility by Polf *et al*.^55^, who studied the radiosensitizing effects of Au nanoparticles. They actually reported an enhancement of proton biological effectiveness of smaller magnitude compared to that experimentally achieved by us. Moreover, cellular survival values measured in our experiments using BSH are essentially identical to those reported by Yasui *et al*.^48^, where DU145 cells were exposed to neutrons in presence of BPA the other commonly used boron-delivering agent).

**Figure 8:**
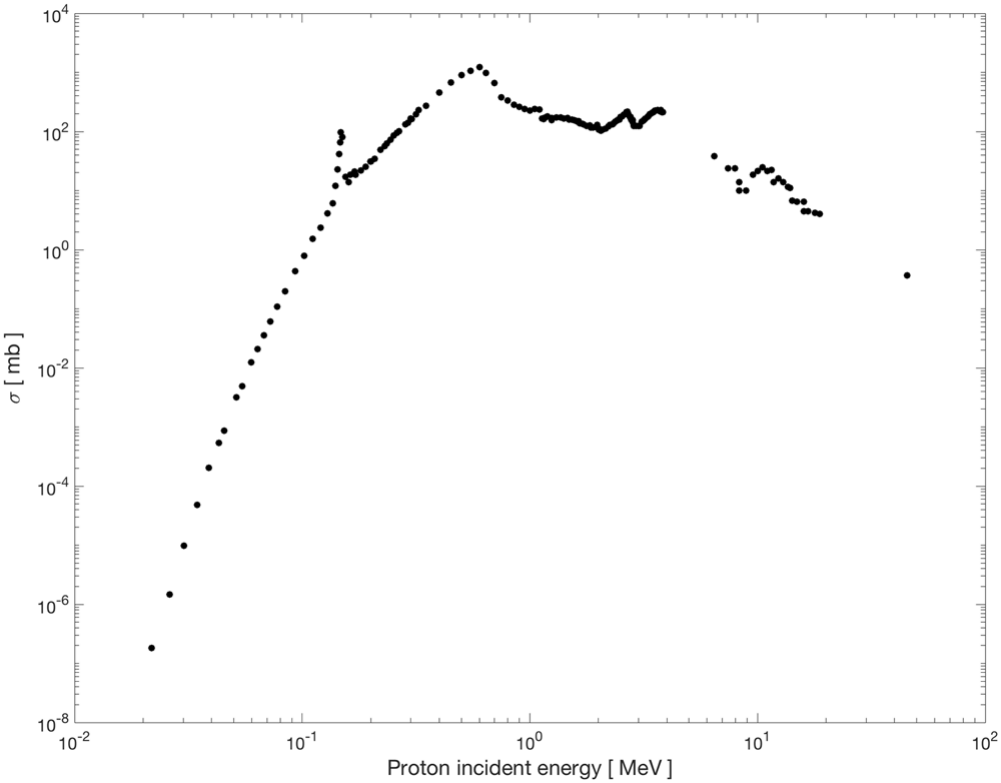
Experimental cross sections. Proton-^11^B total reaction cross section for the most probable α_1_ channel decay (from EXFOR database).

To further consolidate our experimental findings as due to the occurrence of several p-B fusion reaction events, cell survival dose response curves were obtained irradiating DU145 cells at three different positions along the proton SOBP. The results strongly argue in favour of an incident beam energy-dependent increase in proton biological effectiveness since no dose modifying effect could be measured when cells were irradiated in the presence of BSH at the beam entrance, whereas an apparent increase in effectiveness was measured as cells were irradiated at the distal end of the SOBP, in agreement with the cross section of the p-B fusion reaction, which increases with the decrease of mean proton beam energy (see Fig. 8 below).

Investigation of structural DNA damages in the form of CAs not only confirmed that the effectiveness of cellular proton irradiation is enhanced by the presence of boron, as more CAs were found in samples irradiated in the presence of BSH, but the greater occurrence of complex-type exchanges, a well-acknowledged biomarker of high LET radiation exposure, also strongly suggests that such an enhancement could be explained by the action of the high-LET alpha particles. To unveil the potentiating genotoxic action of the boron-carrier BSH two FISH techniques were used: conventional FISH painting and multicolour (m-)FISH. The former allows scoring of chromosome aberration (CAs) types by labelling a limited number of chromosome pairs. By multicolour (m)-FISH, on the other hand, the whole karyotype can be scored thus allowing to detect aberrations that would involve additional chromosomes and would go undetected by the first method. For most purposes, measurement of CA by conventional FISH provides a reliable assessment of radiation-induced genotoxicity. However, mFISH is typically used when a more accurate estimate of complex-type exchanges is needed. Firstly, a greater aberration frequency was found among BSH-treated cells. Following the highest dose of protons used (4 Gy) in the presence of 40 ppm of ^11^B, the observed frequencies of dicentrics (0.144 ± 0.004) and rings (0.064 ± 0.008) are consistent with those of 0.171 ± 0.0175 and 0.029 ± 0.007, respectively, found by Schmid *et al*.^56^ in human lymphocytes at the highest dose of thermal neutrons used by them (i.e. 0.248 Gy) and at a similar ^11^B-equivalent concentration (30 ppm). In addition, a significantly higher proportion of complex chromosome rearrangements in BSH-treated cells compared to controls following proton irradiation was found. This arguably points to an LET-dependent effect since complex CAs are a well-known cytogenetic signature of exposure to high-LET radiation. In fact, it is also known that a greater biological effectiveness of densely ionizing radiation is a direct consequence of the physical pattern of energy deposition events along and around its tracks. Low-LET ionizing radiation such as x- or γ-rays mainly damage cells through short-lived bursts of free radicals (e.g. reactive oxygen species), generated by its interaction with the intracellular environment. This causes isolated lesions at the DNA level, the most detrimental of which for cell survival are double-strand breaks (DSBs). However, the much denser thread of ionization events specific to track-structured high-LET particle radiations, results in many closely spaced clusters of multiply DNA damaged sites, comprising DSBs together with single-strand breaks and damaged bases, which cause the cellular repair system to be error-prone. Hence, such lesions either are left unrepaired, which explains the greater efficiency of high-LET radiations at cell killing, or undergo misrepair. In the latter case, since single densely ionization tracks can simultaneously cause breaks in far apart stretches of DNA, i.e. belonging to separate chromosome domains, defective repair will lead to a higher frequency of DNA mis-rejoining events occurring between several chromosomes compared to the case following low-LET radiation, also known as complex-type exchanges. In BSH-treated irradiated cells, the proportion of such complex-types of aberrations relative to total exchanges ranged between 30% and 42% while in the case of proton irradiation without boron was between 6% and 15%. Moreover, the estimated ratio of complex- to simple-exchanges ranged between 0.59 and 0.71, i.e. similar to value reported in the literature, e.g. 0.64 for 0.5 Gy when only alpha particles were delivered to the biological sample consisting of first-division human lymphocytes scored with conventional FISH painting, i.e. one of the cytogenetic techniques used in our work^57^. Analysis by m-FISH painting corroborated the results obtained by conventional FISH analysis providing an exhaustive and incontrovertible evidence in support of a BSH-assisted increase of proton irradiation –induced complex aberrations.

Although measurements of ^11^B incorporation by cells were not performed, both drug pre-treatment times and concentrations were chosen according to available literature data for the use of the boron-carrier BSH in BNCT, according to which the optimal conditions to reach an ideal boron concentration in cells is 20 µg per grams of tissue or 10^9^ atoms per cell^58^. Indeed, although ^10^B-enriched BSH is known for its poor permeabilization through cell membranes, its use is facilitated by a higher boron content compared to the other widely used boron-delivering compound, such as BPA. However, the alpha particles emitted via the p + ^11^B → 3α reaction present average energies around 4 MeV MeV, which correspond to ranges in tissues of a few tens of microns, thus ensuring a severe cellular DNA damage even if BSH is not incorporated inside the cell but sits on its membrane. Moreover, the ballistic properties of the incident proton beam, whereby proton energies drastically decrease in correspondence with the tumour volume, ensure negligible nuclear fusion events even in the worst-case scenario where the delivery agent containing ^11^B nuclei is also heavily present in the healthy tissues surrounding the tumour. Furthermore, the presence of ^10^B in the proposed method allows triggering various nuclear reactions that generate prompt γ-rays, thus being potentially useful for a simultaneous treatment-and-diagnostics approach^32,43,59^.

The radiobiological data reported in this work suggest that the p + ^11^B → 3α reaction as being responsible for the observed increase in the biological effectiveness of a clinical proton beam. However desirable, we cannot currently provide a simple analytical computation able to explain our results, for instance by correlating the biological effect with the total number of α-particles that can be expected to be generated under our experimental conditions. A possible approach could be trying to calculate the increase in the overall dose and/or LET due to such particles. The current knowledge of biological radiation action has nonetheless established that, the biological effects of low-energy high-LET radiations cannot be interpreted solely on the basis of macroscopic concepts like the absorbed dose or the average LET distributions. This is due to the intrinsically inhomogeneous nature of energy deposition events along radiation tracks, which becomes more significant with their increasing ionization density. Therefore, micro- and nano-dosimetric approaches must be taken into account to analyse the effects arising at cellular level. In addition, the role of extra-targeted phenomena, such as the bystander effect whereby cells that have not been traversed directly by radiation tracks may express cytogenetic damage, is still largely undetermined in such scenario^60^, thereby contributing to the overall uncertainty between the physical dose distribution at the micro or nano-dosimetric level and at the cellular one..

In conclusion, if further confirmed by both *in vitro* and pre-clinical investigations, our results represent an important breakthrough in cancer radiotherapy, particularly in the treatment of such disease by using accelerated proton beams since it may significantly enhance its effectiveness without foreseeable patients’ health complications and added financial costs.

## Methods

### Cell cultures

Prostate cancer cell line DU145 and the spontaneously immortalized, no-transformed human mammary epithelial MCF-10A cells (kindly donated by prof. K. Prise, School of Medicine, Dentistry and Biomedical Sciences, QUB, UK) were grown in 25-cm2 (T25) standard tissue culture flasks, routinely subcultured and maintained at 37 °C in a humidified atmosphere (95% air, 5% CO2). DU145 cells were used to assess possible enhancement by boron of proton-induced cancer cell death while chromosomal DNA damage was investigated in MCF-10A cells. DU145 cells were grown in RPMI medium supplemented with 10% fetal bovine serum and 1% antibiotics. Two media were instead needed for MCF-10A cells, one for optimal growth conditions and the other for resuspension and quenching of trypsin, as described in detail by Debnath *et al*.^61^. Two days before irradiation DU145 and MCF-10A cells were seeded in T25 flasks at 10^5^ and 6 10^5^ cells/flask, respectively.

### BSH preparation and treatment

A 1-g vial of sodium mercaptododecaborate or N-BSH, Na_2_ [B_12_H_11_SH], FW 219.87, was purchased from KATCHEM Ltd. (Czech Republic). The working concentrations of 80 and 40 ppm of ^11^B corresponded to 0.17 mg/ml and 0.08 mg/ml of BSH, respectively. BSH was decanted at the necessary amounts according to the total volume of the medium, in which the compound was thoroughly dissolved by simple agitation just prior to cell treatment. Boron cellular conditioning started 7 hrs prior to irradiation: the cell growth medium was aspirated and replaced with 5 ml of BSH-containing medium. Ordinary BSH-free growth medium was replaced into flasks that were used as controls. Just before irradiation, flasks were completely filled with the respective media (with or without BSH) to prevent cells from drying since flasks are irradiated standing vertically in front of the beam.

### p + ^11^B → 3α nuclear fusion

The p + ^11^B → 3α nuclear fusion reaction at low energy can be basically described as a two-step reaction due to its behaviour at the three resonant energies (0.162 MeV, 0.675 MeV and 2.64 MeV). Firstly, a proton, interacting with ^11^B, induces the formation of a ^12^C* compound nucleus formed in the 2- or 3- excited state. If the ^12^C* nucleus is formed in its 2- state, it will decay to the first 2+ stat of ^8^Be emitting one alpha-particle with l = 3^62^. If the ^12^C* nucleus is formed in its 3- state, then the primary alpha particle can be emitted either with l = 1 from the decay to the first 2+ ^8^Be excited state, or with l = 3 from the decay to the 0 + 8Be ground state. In either case, the remaining ^8^Be (2+ or 0+) nucleus immediately decays into two secondary alpha particles with l′ = 2. Alpha particles emitted in the first stage present a well-defined energy distribution and are commonly referred to as α0 and α1 if the ^8^Be 2+ or the 0+ states are populated, respectively. Few authors^62,63^ report that a very unlikely fourth channel, characterized by a maximum cross section of 10 μb in the 2.0–2.7 MeV energy range, can also be activated. In this case the ^12^C* directly breaks into three α particles skipping the intermediate ^8^Be stage, which show a continuous energy distribution.

Figure 8 reports some of the available experimental data^62,63^ for the total production cross section of the p + ^11^B → 3α reaction as function of the mass centre energy and for the α_1_ channel. For low energies (0.1–5 MeV) the reaction cross sections become significantly high, thus maximising the alpha particle production around the proton Bragg peak region, an advantageous feature for the alternative protontherapy approach proposed in this work.

### Irradiation

Irradiations were performed using the 62-MeV proton beam generated by the superconducting cyclotron clinically used at the CATANA (Centro di AdroTerapia ed Applicazioni Nucleari Avanzate)^64,65^ eye proton therapy facility of the Italian Institute for Nuclear Physics in Catania, Italy. The CATANA irradiation setup for biological samples is described in detail elsewhere^65^. The clinical Spread Out Bragg Peak (SOBP) range was 30 mm in water and cells were positioned at the depth of 24.86 mm water equivalent (calculated incident LET ~ 5 keV/μm), to the middle of the SOBP. Absolute absorbed dose is measured in water, by means of a plane-parallel PTW 34045 advanced-type Markus ionisation chamber, according to the International Atomic Energy Agency Technical Report Series 398 Code of practice^66^. The absorbed dose in water per monitor unit (cGy/M.U.) for the specific modulated beam used to irradiate the cells was measured at the isocenter. The dose measurements were carried out at the depth corresponding to the middle of the SOBP, using a reference 25 mm diameter circular collimator^65^. The clinical proton beam calibration was performed just before each irradiation; the variation of beam calibration on the various experiments resulted to be within 3%. Overall uncertainty in absolute dose measurement is kept within 1.5%. Details on the irradiation beamlines, dosimetric procedures and related uncertainties for irradiation conditions can be found elsewhere^66^. For measurement of cell killing, proton doses of 0, 0.5, 1, 2, 3 Gy were used. For 80 ppm of ^11^B the effects of a dose of 4 Gy were also tested. One cell culture flask was used for each dose for all BSH concentrations in each experiment. For chromosome aberration studies, three doses were used: 0.5, 2 and 4 Gy. Two flasks per dose were used for all BSH concentrations. Four independently repeated experiments were performed for clonogenic survival with protons alone and 80 ppm of ^11^B, and two independent experiments for clonogenic cell death with 40 ppm ^11^B. Two independent experiments were carried out for chromosome aberration analysis.

In the case of measurement of clonogenic survival along the proton SOBP, appropriate stacks of PMMA degraders were realised to achieve varying depths along the dose-depth profile using the same setup as described in Chaudray *et al*.^14^. Low-LET reference irradiations were carried out at Physics Department, University of Naples Federico II, employing a 250 kV_p_ radiogen x-ray STABILIPAN machine (Siemens), with 1-mm Cu filter and a dose rate of about 1.2 Gy min^−1^.

### Measurement of cell death

Radiation-induced cell death was assessed by means of the clonogenic test, the gold standard for measuring cellular radiosensitivity. According to this assay, a cell survives irradiation if it retains its proliferative potential and it is thus capable of forming a colony composed of at least 50 cells. After irradiation, the medium was discarded from the flasks, and DU145 cells were detached, counted by haemocytometer and re-plated at opportune densities. They were then allowed to grow for colony formation in BSH-free medium. Four replicates for each dose were used for statistical data robustness. Clones were fixed and stained by crystal violet after 10 days. Surviving fractions (SF) are obtained by dividing the number of clones by the number of cells seeded at a given dose D, normalized by the plating efficiency (PE), that is the “surviving” of cells in the absence of radiation according to the expression SF_DoseD_ = [Number of clones/number of cells]_DoseD_/(PE). Experimental data were fitted to the linear-quadratic equation that best describes low-LET radiation induced cell death SF = exp (-α*D-β*D^2^).

### Chromosomes aberration analysis

After irradiation, MCF-10A cells were kept in BSH-free medium for up to 36 h. Genotoxic action of radiation was studied by scoring structural aberrations in chemically induced interphase chromosomes according to the Premature Chromosome Condensation (PCC) technique. Cells were incubated for 30 min with calyculin A (50 ng/mL, Sigma-Aldrich) for PCC induction as elsewhere described^67^. To collect PCC spreads, cells were trypsinized, centrifuged at 300g for 8 min, then the pellet was resuspended for 25 min in hypotonic solution (75 mM KCl at 37 °C), and fixed on ice in freshly prepared Carnoy solution (3:1 v/v methanol/acetic acid). Spreads were then dropped on pre-warmed (42 °C) wet slides and air-dried at room temperature. Conventional Fluorescence *In Situ* Hybridization (FISH) painting was conducted by using whole-chromosome fluorescence-labelled DNA painting probes directed to chromosomes 1 and 2 following the manufacturer’s recommendations (MetaSystems, Germany). Denaturation (72 °C for 3 min) followed by hybridization (37 °C overnight) of slides was performed using a hand-free HYBrite chamber system. Unlabeled chromosomes were counterstained with 12 ml 4, 6-diamidino-2-phenylindole (DAPI) staining. For m-FISH, the 24XCyte probe by MetaSystems was applied to chromosomes harvested as above described. The labelling kit contains five fluorochromes:

- Cy^TM^5 - Cyanine 5, fluorescing in the red region (649 nm excitation, 670 nm emission);
- DEAC (DiEthylAmino-Coumarin) - λ_ex_ = 409 nm, λ_em_ = 473 nm;
- FITC (Fluorescein IsoThioCyanate) - λ_ex_ = 495 nm, λ_em_ = 525 nm;
- Spectrum Orange^TM^ - λ_ex_ = 554 nm, λ_em_ = 587 nm;
- Texas Red^®^ - λ_ex_ ~ 596 nm, λ_em_ ~ 620 nm.

Software ISIS^®^ imaging system (MetaSystems) attributes a false color pattern depending on overlap signals intensity, according to 24XCyte labeling scheme shown below (Table 3).

**Table 3:**
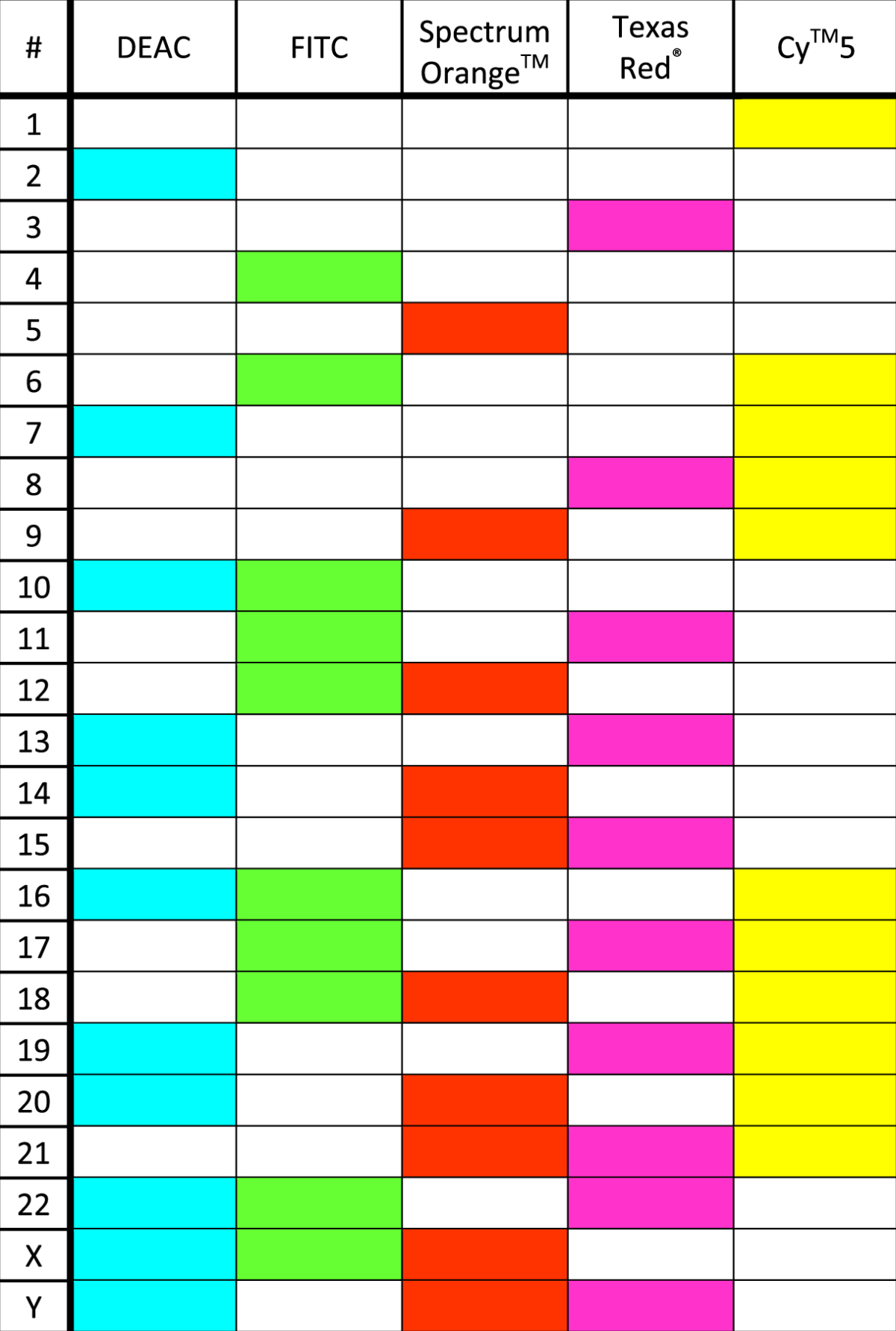
24Xcyte labelling scheme whereby each chromosome is labelled by the combination of the main five fluorochromes.

As per manufacturer’s directions, chromosomes and probe were denaturated by sequential treatment by 2X Saline-Sodium Citrate (SSC) solution at 70 °C for 30 min, followed by room temperature (RT) bathing in 0.1x SSC for 1 min, by RT rinse in 0.07 NaOH for 1 min, 4 °C wash in 0.1X and 2X SSC for 1 min each. Final step was sequential washing in ethanol series (70%, 95% and 100%). While slides air-dried, probe was denaturated by incubating at 75 °C incubation in a water bath for 5 min, ice-cold cooling for 30 min and incubation at 37 °C for 30 min. Denatured probe was then applied to the slide (12 μl for a 24 × 24 mm^2^ coverslip). Hybridization was thus carried out for 48 h using the above-mentioned HYBrite chamber. After post-hybridization washing (0.4X SSC, pH 7.0–7.5, at 72 °C; RT 2X SSC, pH 7.0–7.5, containing 0.05% Tween20®), DAPI/antifade (250 ng/ml) counterstaining was applied.

For conventional FISH scoring, slides were viewed with an epi-fluorescence microscope connected to a computer-controlled system (Metafer 4 software, MetaSystems), for automated slide scanning and three-color image acquisition. Chromosome analysis was carried out on stored images. Scoring was conducted blind by the same scorer. Not less than 500 chromosome spreads for each dose were scored (at least 1,500 for nonirradiated controls). Karyotype reconstruction and analysis was manually carried out using an Axio Imager M1 fluorescence microscope (Zeiss) with the help of the ISIS software for image processing. At least 200 chromosome spreads were analyzed for each experimental point. All types of aberrations were scored separately and categorized as simple exchanges (i.e. translocations and dicentrics), either complete or incomplete, acentric fragments and complex exchanges ^68^. For this study’s purpose, however, data herein presented are relative to the total chromosomal exchange frequency and to the complex-type exchange frequency. Frequency of total aberration exchanges was fitted to the equation Y = Y_0_+α*D+βD^2^.

### Statistical analysis

For analysis of the dose – response relationships for cell killing and chromosome aberration frequency, curve fitting was performed by nonlinear least square minimization (Marquardt’s algorithm) using SigmaPlot 12.5 software (SPSS, USA). Poisson statistics was assumed to derive standard errors on aberration frequencies. Experimental surviving fraction data in clonogenic assays are affected by several sources of errors, such as those associated with cell counts and cell dilutions, which are not taken into account by simple calculations of the standard error affecting colony counting. A more precise approach is needed to determine the experimental error on the plating efficiency PE as above defined and here recalled for convenience:

1 $$PE=(\frac{{\bar{X}}_{colonies}}{{\bar{X}}_{cells}})|0\,Gy$$

This quantity is the ratio between two mean values, i.e. the mean counted colony number and the mean number of cells seeded, each with its standard error SE: SE_colonies_ and SE_cells._ Therefore, according to the standard formula on error propagation, the standard error for plating efficiency, i.e. SE (PE), can be derived as follows:

2 $$\begin{array}{rcl}SE(PE) & = & \sqrt{{(\frac{\partial PE}{\partial {\bar{X}}_{colonies}})}^{2}{(S{E}_{colonies})}^{2}+{(\frac{\partial PE}{\partial {\bar{X}}_{cells}})}^{2}{(S{E}_{cells})}^{2}}\,\\  & = & \sqrt{{(\frac{1}{{\bar{X}}_{cells}})}^{2}{(S{E}_{colonies})}^{2}+{(-\frac{{\bar{X}}_{colonies}}{{{\bar{X}}_{cells}}^{2}})}^{2}{(S{E}_{cells})}^{2}}\end{array}$$

Recalling the definition of Surviving Fraction at a given dose D:

3 $$S{F}_{D}=(\frac{{\bar{X}}_{colonies}}{{\bar{X}}_{cells}})/PE$$

In the interest of simplicity, it can be assumed that the mean number of cells seeded that appears in the above formula is devoid of error, which is reasonable since cell counting error is taken care of in the SE (PE). This means treating ${\bar{X}}_{cells}$ as a constant; hence we can define $\frac{{\bar{X}}_{colonies}}{{\bar{X}}_{cells}}$ as SF, with its error being (SE_colonies_)/$(\,{\bar{X}}_{cells})$.Hence, SE(SF_D_) can be determined as follows:

4 $$SE(S{F}_{D})=\,\sqrt{{(\frac{1}{PE})}^{2}{(\frac{S{E}_{colonies}}{{\bar{X}}_{cells}})}^{2}+{(-\frac{SF}{{(PE)}^{2}})}^{2}{(SE(PE))}^{2}}$$

In these calculations, we assumed that observed CV is always greater than Poisson CV on all counts, of either colonies or cells; wherever this was no the case, we corrected the experimental SE by multiplying the mean (of cell or colony counts) by the Poisson CV.Error on calculated DMF at 10% survival (SF) level defined as:

5 $$DM{F}_{10}=\,(\frac{{D}_{noBSH}}{{D}_{BSH}})|SF=0.1\,$$

was derived by using error propagation as given by:

6 $$\begin{array}{rcl}\sigma DM{F}_{10} & = & \sqrt{{(\frac{\partial DM{F}_{10}}{\partial {D}_{noBSH}})}^{2}{({\sigma }_{{D}_{noBSH}})}^{2}+{(\frac{\partial DM{F}_{10}}{\partial {D}_{BSH}})}^{2}{({\sigma }_{{D}_{BSH}})}^{2}}\\  & = & \sigma DM{F}_{10}=DM{F}_{10}\,\sqrt{{(\frac{\sigma {D}_{noBSH}}{{D}_{noBSH}})}^{2}+{(\frac{\sigma {D}_{BSH}}{{D}_{BSH}})}^{2}\,}\end{array}$$

Dose uncertainties were derived using error propagation from the linear-quadratic equation SF = exp (−α*D-β*D^2^), which in its most general formulation reads as:

7 $${\sigma }_{D}=\,\sqrt{{(\frac{\partial D}{\partial \alpha })}^{2}{\sigma }_{\alpha }^{2}+{(\frac{\partial D}{\partial \beta })}^{2}{\sigma }_{\beta }^{2}}$$

Rigorously, in formula 7 there is also the covariance of the parameters α and β, which, for clonogenic survival curves, is negative as they are weakly anti-correlated, hence, this term is neglected to avoid underestimation of the error, with the partial derivatives of the dose with respect to α and β being

8 $$\frac{\partial D}{\partial \alpha }=\,-\frac{D}{\alpha +2\beta D}$$

9 $$\frac{\partial D}{\partial \beta }=-\frac{{D}^{2}}{\alpha +2\beta D}$$
